# A Biased Competition Theory of Cytotoxic T Lymphocyte Interaction with Tumor Nodules

**DOI:** 10.1371/journal.pone.0120053

**Published:** 2015-03-27

**Authors:** Claire Christophe, Sabina Müller, Magda Rodrigues, Anne-Elisabeth Petit, Patrick Cattiaux, Loïc Dupré, Sébastien Gadat, Salvatore Valitutti

**Affiliations:** 1 Institut de Mathématiques de Toulouse, CNRS UMR 5219, Toulouse, France; 2 INSERM, UMR 1043, Centre de Physiopathologie de Toulouse Purpan, Section Dynamique Moléculaire des Interactions Lymphocytaires, Toulouse, France; 3 Université Toulouse III Paul Sabatier, Toulouse, France; 4 Ludwig Institute for Cancer Research, Brussels Branch, Université de Bruxelles, Bruxelles, Belgique; 5 Toulouse School of Economics, Université de Toulouse I Capitole, Toulouse, France

## Abstract

The dynamics of the interaction between Cytotoxic T Lymphocytes (CTL) and tumor cells has been addressed in depth, in particular using numerical simulations. However, stochastic mathematical models that take into account the competitive interaction between CTL and tumors undergoing immunoediting, a process of tumor cell escape from immunesurveillance, are presently missing. Here, we introduce a stochastic dynamical particle interaction model based on experimentally measured parameters that allows to describe CTL function during immunoediting. The model describes the competitive interaction between CTL and melanoma cell nodules and allows temporal and two-dimensional spatial progression. The model is designed to provide probabilistic estimates of tumor eradication through numerical simulations in which tunable parameters influencing CTL efficacy against a tumor nodule undergoing immunoediting are tested. Our model shows that the rate of CTL/tumor nodule productive collisions during the initial time of interaction determines the success of CTL in tumor eradication. It allows efficient cytotoxic function before the tumor cells acquire a substantial resistance to CTL attack, due to mutations stochastically occurring during cell division. Interestingly, a bias in CTL motility inducing a progressive attraction towards a few scout CTL, which have detected the nodule enhances early productive collisions and tumor eradication. Taken together, our results are compatible with a biased competition theory of CTL function in which CTL efficacy against a tumor nodule undergoing immunoediting is strongly dependent on guidance of CTL trajectories by scout siblings. They highlight unprecedented aspects of immune cell behavior that might inspire new CTL-based therapeutic strategies against tumors.

## Introduction

CTL destroy virally infected cells and tumor cells via the secretion of lytic molecules stored in intracellular granules [1]. CTL are key components of the anti-cancer immune response and it is therefore crucial to study in depth, and possibly enhance, their biological responses against tumors [2]. Accordingly, therapeutic protocols designed to potentiate CTL responses against tumor cells are currently at the frontline of cancer clinical research [3]. The molecular mechanisms of tumor recognition by CTL and the biological responses of CTL against tumors have been thoroughly investigated. However, since CTL/tumor cell interactions are highly dynamic, it is crucial to define the cell motility and interaction parameters that might influence CTL efficacy against tumor cells and tumor eradication.

Limited tumor site accessibility by CTL and intrinsic tumor cell resistance to CTL attack are major limits to CTL-mediated immune surveillance [4]. Moreover, the tumor micro-environment can progressively affect CTL function, leading to CTL exhaustion [5]. Finally, it has been demonstrated that the adaptive immune response plays a dual role in cancer. While CTL can control tumor growth by destroying tumor cells, the selective pressure of the immune system promotes tumor progression by selecting tumor variants that are fit to survive in an immunocompetent host. Such a process is defined as cancer immunoediting [6]. The design of immunotherapies that could bypass cancer immunoediting is presently a major conundrum in cancer clinical research. Theoretically, one possibility would be to facilitate the access of CTL to the tumor site in order to eradicate the tumor before immunoediting can occur. In this context, an attractive hypothesis is that CTL might serve as scouts of their siblings. In agreement with this hypothesis, it has been shown that CTL can rapidly release the CTL-attracting chemokine CCL5 upon TCR productive engagement [7]. Whether chemotactic responses of CTL to chemokine released by other CTL having contacted the tumor nodule could be exploited in the context of antitumor therapeutic strategies remains to be elucidated.

Experimental approaches aiming at recapitulating the complex evolving balance between CTL efficacy and the resistance of a multicellular tumor nodule are technically difficult to perform. Moreover, the integration of the parameters influencing this balance, in the context of hypothetical human tumors, leads to a very complex dynamical predator/prey system [8].

Here we propose a computational model based on experimental measurements that provides a comprehensive view of the kinetic parameters of such a predator/prey system [8]. We present a stochastic dynamical model and numerical simulations that describe the competition between spherically growing tumors and a clonal population of CTL, model that will be accurately described below. We focus on melanoma since in this neoplastic disease, tumor-associated antigens have been described and are known to elicit CTL-based immune responses that are counteracted by immunoediting processes in the tumor microenvironment [9, 10]. Moreover various CTL-based adoptive transfer therapies are currently under evaluation for melanoma patients [11]. We consider here only planar interactions. This is a simplification, yet it has the advantage of allowing a clear representation of cellular interactions and a rapid numerical implementation of the model in order to vary different parameters of CTL function and tumor growth either individually or simultaneously. Moreover the 2-D model allows to incorporate *in vitro* microscopy data that were acquired in 2-D. It has been previously shown that the kinetic evolution of tumor growth and CTL displacement follows a stochastic dynamics [12, 13]. We therefore adopt a stochastic modeling rather than a deterministic evolution of CTL displacement, killing capacity and cancer immunoediting.

Our results show that in a context of stochasticly occurring mutations leading to tumor immunoediting, CTL attraction towards scout siblings having detected the tumor is a crucial parameter allowing early productive CTL/tumor collisions and therefore tumor eradication. We propose a biased competition theory of CTL function in which a biased displacement of CTL is instrumental for the efficacy of their effector function.

## Description of the model

The theoretical model presents the stochastic evolution of a growing tumor undergoing immunoediting, facing motile CTL that have the capacity to kill tumor cells. The model provides a basis to run multiple simulations, which are used to compute the probability of success/loss in tumor eradication. All the experimental parameters used for the numerical simulations of the model are issued from [14] or have been measured by us in this study (Supporting Information S1 Table lists the parameters used in the model).

### Cell behaviors

Before a description of each theoretical evolution, we first enumerate the several stochasticized behaviors taken into account in our model.

**Fig 1:**
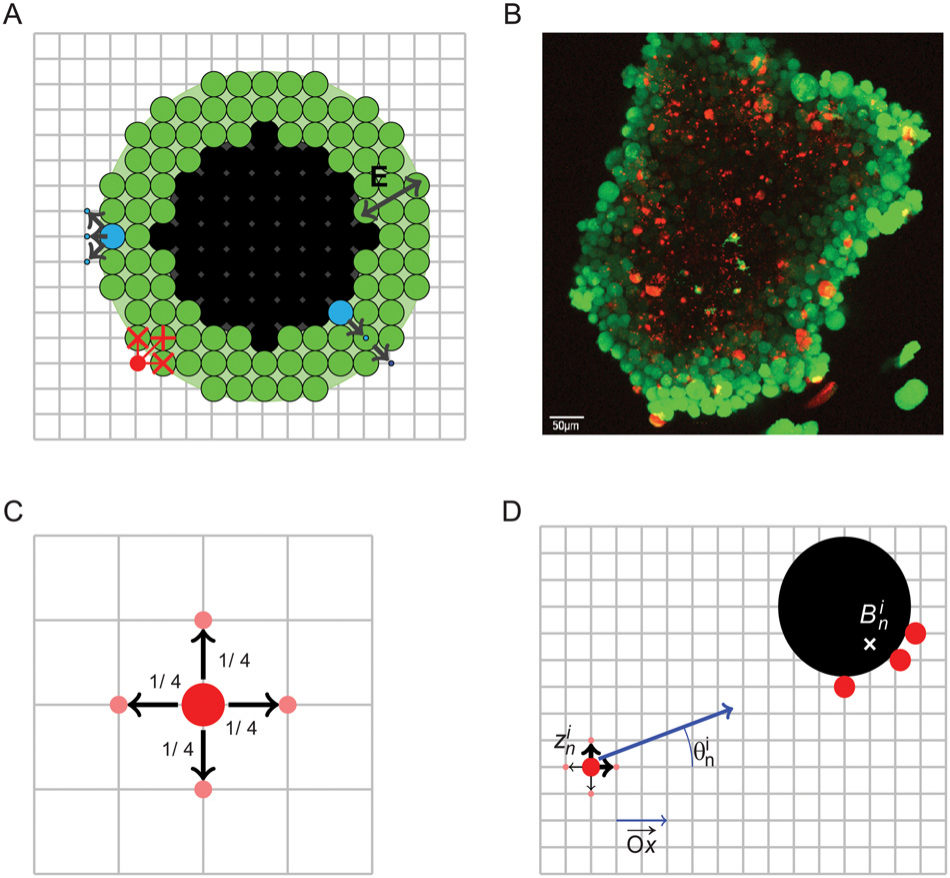
Graphical representation of the model. (*A*) Synthetic planar structure of the nodule, fitted to the grid. Green: cells in the proliferative shell. Black: non proliferative and necrotic cells.: CTL killing its $\sqrt{2}$-neighborhood tumor cells. Blue: two tumor cells attempt to divide. On the left, a tumor cell is dividing towards one of the three free available positions (black arrows). On the right, there is no free location available at the time of division, the dividing cell pushes its close neighbors towards the exterior of the nodule. (*B*) Picture of a melanoma nodule. Green: alive cells, red: dead cells. (*C*) Random walk on the 2D grid of one CTL. Red: position of the CTL. Black arrows: admissible displacements. (*D*) One CTL at position $z_{n}^{i}$ is attracted by 3 scout sibling CTL (in red) that have hit the tumor nodule (black sphere). The barycenter $B_{n}^{i}$ is the target position for $z_{n}^{i}$. The random walk of CTL on position $z_{n}^{i}$ is reinforced towards $B_{n}^{i}$ (blue arrow), please see material and methods section for further details.

- Since we aim to describe the competition between the tumor nodule and the CTL, we consider a longitudinal section of the tumor nodule evolving on a compact two-dimensional domain described as a grid Ω, in parallel with a population of motile CTL. The dimension of the considered grid Ω is constant. At the beginning of the evolution, Ω is much larger than the tumor. This assumption can become false along the temporal evolution when the population of CTL fails to eradicate the tumor, leading to uncontrolled tumour nodule growth.
- Each CTL moves according to a symmetric random walk on Ω and is reflected when it eventually reaches the boundaries of the grid. This is an important assumption for our stochastic model. We believe that this assumption is legitimate since it describes the fact that even if some CTL can exit Ω in a real biological framework, other CTL can enter, keeping the size of the CTL population constant. This last assumption could be amended by considering a CTL population whose size can be modified over time, through an unbalance between CTL entering and exiting Ω, however, we have not pushed further our investigations in this direction.
  Hence, this shortcut roughly means that we will consider a population of CTL of constant size. When a CTL encounters a tumor cell, it automatically stops its walk and starts the killing process of the tumor cell, which is also stochastic according to our model. We propose two displacement models in what follows: the first one is a pure symmetric random walk already described in [13]; the second is a self-interacting re-inforced random walk, whereby CTL are attracted towards scout siblings that have encountered the tumor nodule.
- All along the computational time, a CTL becomes “exhausted” within the tumor environment when it reaches a maximal number of 5 killing rounds. Hence, an exhausted CTL can kill no more tumor cells. This value is based on our expertimental observations under *in vitro* conditions in which human CTL kill ∼ 5 target cells over prolonged time. Exhausted CTL also evolve in Ω according to a random walk, bouncing back both when encountering the boundaries of the grid and when contacting the tumor mass. CTL exhaustion is irreversible since CTL are confined within the tumor microenvironment and therefore they cannot reacquire an activation status. This parameter has been inserted in the model in order to mimic the *in vivo* situation in which tumor infiltrating CTL exhibit an exhausted phenotype and are functionally impaired [5].
- The tumor nodule is composed of 2 different areas: the proliferative shell composed of actively dividing cells and the central core composed of non-proliferative cells (See Fig. 1*A*). This concentric (but not necessarily exactly spherical structure) mimics the structure of tumor clusters as defined by confocal laser scanning microscopy (See Fig. 1*B*). Without CTL intervention, the proliferative shell will stochastically grow, with a dynamic described by a Galton-Watson branching process (each cell of the proliferative shell is divided in 2 cells after a stochastic geometrical time, see details below).
- In the model we implement the possibility that, along each division, tumor cells could acquire mutations that might render them progressively more resistant to CTL attack (as part of an immunoediting process) or even invisible to CTL (mimicking MHC Class I loss described in tumor cells during immunoediting [6]). Hence, for the acquisition of resistance, daughter tumor cells can become stochastically and progressively more resistant than their mother cells.

### Discretization of the time and of Ω

CTL and tumor cells are spatially parameterized by their coordinates (*x*, *y*) on a discrete grid (See Fig. 1*A*), where the step size movement Δ of each CTL is

$$\begin{array}{c} |(x,y)-(x+1,y)|=|(x,y+1)-(x,y)|=12.5\mu m=\Delta , \end{array}$$

this value corresponds to the mean diameter of a melanoma cell as measured by confocal laser scanning microscopy. The evolution is discretized according to a time step *δ*_*t*_, so that the time sequence (*t*_*n*_)_*n* ≥ 0_ is equally spaced: *δ*_*t*_ = *t*_*n*_ − *t*_*n*−1_. The time step *δ*_*t*_ is fixed to 1.44 minutes (see material and methods) according to experimental measurements of the mean CTL velocity (the mean recorded velocity is about 8.66*μm*/*min*). Such settings make it possible to handle CTL movements on the grid (discretized on the basis of the size of a tumor cell) with a velocity that reproduces experimental measurements.

### Movement of the CTL population

The number of CTL (*N*_*CTL*_) is fixed for each numerical simulation, assuming that cells entering in the field or generated by cell division replace CTL leaving the field or dying. For CTL displacements, positions are parameterized by their planar coordinates at time $t_{n}:\;z_{n}^{i}:=(x_{n}^{i},y_{n}^{i})$, for *i* = {1, …, *N*_*CTL*_} and these coordinates still belong to the regularly spaced grid. Without any interaction with the tumor nodule or another CTL, each CTL evolves according to a symmetric random walk moving from one location to another to its 1-neighbors (see Fig. 1*C*). Equation (1) describes the displacement probability of each CTL *i* in the grid in the four directions of the space:

$$\begin{array}{ccc} P\left[z_{n+1}^{i}=z_{n}^{i}+\left(1,0\right)\right] & = & P\left[z_{n+1}^{i}=z_{n}^{j}+\left(-1,0\right)\right]\\ & = & P\left[z_{n+1}^{i}=z_{n}^{i}+\left(0,1\right)\right]\\ & = & P\left[z_{n+1}^{i}=z_{n}^{i}+\left(0,-1\right)\right]=1/4. \end{array}$$ (1)

We also propose a second displacement model based on a self-interacting re-inforced random walk.

### Biased displacement of CTL through chemotactism

In the attempt to optimize the number of CTL collisions with tumor cells, we introduced a bias for CTL movement. The updated model postulates that when a CTL collides with the tumor nodule, it releases chemo-attractants that guide the trajectories of other CTL towards the nodule (see Fig. 1*D* for a graphical illustration). We thus consider a self-interacting particle system, inspired from population dynamics already described in other biological fields (e.g. ant colonies [15]). The principle can be described in this way: when one (or several) CTL hits the boundary of the nodule, other CTL may be attracted to the position of this scout CTL if they are located close enough (the distance between scout cells and attracted ones should be less than a parameter D). In these conditions, the model postulates that a given CTL *i* (whose position $z_{n}^{i}$ does not belong to the frontier of the nodule at time *t*_*n*_) is attracted towards the barycenter of the scout CTL $B_{n}^{i}$ (see Fig. 1*D*). If $\theta_{n}^{i}$ denotes the planar angle between the two vectors $\vec{B_{n}^{i}z_{n}^{i}}$ and $\vec{Ox}$ (as shown in Fig. 1*D*), the attraction towards the barycenter is provided by $\nu_{n}^{i}$ that belongs to [0, *ν*], which quantifies the attraction strength. This parameter $\nu_{n}^{i}$ depends on the distance between $B_{n}^{i}$ and $z_{n}^{i}$, and on the number of scout CTL, denoted $K_{n}^{i}$. The model postulates a fixed maximal scout CTL number *K* (corresponding to 10 CTL) and a maximal attraction strength *ν*. To define the impact that the attraction of CTL towards the tumor nodule might have on tumor eradication, we let vary the parameter *ν* in the different numerical simulations. Finally, we use the following formula

$$\begin{array}{c} \nu_{n}^{i}=\left(1-\frac{|z_{n}^{i}-B_{n}^{i}|}{D}\right)\times \min\left\{1;\frac{K_{n}^{i}}{K}\right\}\times \nu , \end{array}$$

which stands that the maximal attraction number is reached when $K_{n}^{i}=K=10$ CTL and the attracted CTL $z_{n}^{i}$ is very close to the barycenter $B_{n}^{i}$.

Thus, the position of $z_{n+1}^{i}$ (CTL moves from $z_{n}^{i}$ to its 1-neighbors) follows the mixture law $(1-\nu_{n}^{i})\times$ symmetric random walk $+\nu_{n}^{i}\times$ drifted attraction towards $B_{n}^{i}$. Precisely, if *ϵ*(*a*) denotes the algebraic sign of any real number *a*, we define

$$\begin{array}{c} P\;\left[z_{n+1}^{i}=z_{n}^{i}-\left(\epsilon \left(\cos\theta \right)\right),0)\right]=P\;\left[z_{n+1}^{i}=z_{n}^{i}-\left(0,\epsilon \left(\sin\theta \right)\right)\right]=\frac{1-\nu_{n}^{i}}{4} \end{array}$$

and

$$\begin{array}{c} P\;\left[z_{n+1}^{i}=z_{n}^{i}+\left(\epsilon \left(\cos\theta \right)\right),0)\right]=\frac{1-\nu_{n}^{i}}{4}+\nu_{n}^{i}\cos{\left(\theta \right)}^{2}, \end{array}$$

as well as

$$\begin{array}{c} P\;\left[z_{n+1}^{i}=z_{n}^{i}+\left(0,\epsilon \left(\sin\theta \right)\right)\right]=\frac{1-\nu_{n}^{i}}{4}+\nu_{n}^{i}\sin{\left(\theta \right)}^{2}. \end{array}$$

### CTL killing

A single CTL detects the nodule through a contact process: when one CTL hits the frontier of the nodule, the CTL stops moving instantaneously and starts the killing of the tumor cells in its immediate $\sqrt{2}-$neighborhood (Fig. 1*A*). We assume that before exhaustion, a CTL kills a tumor cell with a constant rate *μ*, which is transposed in our model by a continuous exponential distribution for killing time. According to our time discretization, such a probabilistic distribution needs to be slightly modified. The distribution of the stochastic killing time is thus converted to a stochasticly geometrical law 𝓖(μ*) which is the equivalent distribution for discrete time to an exponential law in a continuous setting.

The mean measured time in minutes for detection of apoptosis induction in melanoma cells *in vitro* [14] is 1/*μ* = 26.4*min*. To obtain the inverse of the mean time required for killing one target cell according to the discretized time used in simulations, it is necessary to compute μ* = *μ* ⋅ *δ*_*t*_ (see material and methods). Hence, the probability that a CTL *i* kills a tumor cell at time *t*_*n*_ is

$$\begin{array}{c} P\;\left(T_{kill}^{i}=t_{n}\right)=\mu^{*}{\left(1-\mu^{*}\right)}^{n-1}. \end{array}$$

After killing a first tumor cell, the CTL restarts a symmetric random walk according to the rules defined in (1). A CTL becomes “exhausted” when it reaches a maximal number of killing rounds fixed to 5.

### Tumor nodule growth

In the proliferating shell, tumor cells are divided according to a stochastic time that follows a geometric distribution. The average measured time of duplication of melanoma cells *in vitro* is 16.66 hours (1000*min*, as we experimentally measured). This is used to fix the tumor division rate parameter via λ* = *λ* × *δ*_*t*_, where 1/*λ* = 1000 (see material and methods). The model assumes that the probability for a tumor cell *i* to divide at a time *t*_*n*_ is

$$\begin{array}{c} P\;\left(T_{i}^{Div}=t_{n}\right)=\lambda^{*}{\left(1-\lambda^{*}\right)}^{n-1}. \end{array}$$

The tumor nodule growth is also described by the parameter *E* (*E* = thickness of the proliferating shell) (see material and methods). It means that per unit time, the dividing rate of tumor cells in the proliferative shell (corresponding to the cells for which the distance from the nodule frontier is lower than *E*) is λ*. Each division yields the addition of a new tumor cell. Two cases are possible:

1. Some free location is available in the immediate neighborhood ($\sqrt{2}$ -neighborhood) of the mother cell and the new cell chooses its place uniformly among all free neighborhood;
2. The new cell chooses an occupied location in its $\sqrt{2}$-neighborhood and pushes another cell located in the proliferating shell towards the exterior of the nodule. Recursively, if the pushed cell encounters another tumor cell, it pushes this cell towards the exterior of the nodule and so on. Equivalently, when a cell in the interior of the proliferative part divides, it adds a new cell on the closest point of the frontier of the nodule.
  Both cases of such dynamics are illustrated in Fig. 1*A*. After each division event, the proliferative shell is instantaneously updated.

### Immunoediting

As pointed above, tumor cells could acquire mutations and then become resistant to CTL lysis. We consider that after each duplication of a tumor cell, a daughter cell could become progressively more resistant with a rate *p*_*res*_. In this case a CTL will kill daughter cells within a time longer than that required to kill mother cells. The model postulates several levels of resistance *R* ∈ {1, 2, 3, …}. A tumor cell with *R* = 1 is a tumor cell which did not increase its resistance upon division. A tumor cell with *R*_*d*_ = *R*_*m*_ + 1 is a tumor cell which increases its resistance upon one division (*R*_*d*_ is the resistance of the daughter cell, *R*_*m*_ is the resistance of the mother cell). As a result, each tumor cell acquiring resistance upon division requires on average twice as long as its mother cell to be killed, yet it can still be killed. In the model, a tumor cell becomes invisible to CTL with the rate *p*_*inv*_. We assume that the probability for a tumor cell to become invisible (*p*_*inv*_) is equal to 0 if the tumor cell has not acquired resistance (*R* < 3). This probability is about $\frac{1}{12}$ if *R* = 3 and increases with the resistance of the mother cell up to $\frac{1}{4}$. See Supporting Information S1 Table for the values of *p*_*res*_ and *p*_*inv*_.

### Stochastic dynamical agent based model or differential equations?

Computational works on biological dynamical systems consider either infinitesimal agent based model described by a stochastic particles system or partial differential equations (PDE) that describe the mean chronological evolution of a macroscopic number of interest (density of CTL, size of the tumor nodule, …). Indeed, these two alternative ways of describing the biological system are generally equivalent. On the one side it is easier to amend the dynamical model with particles systems because of its flexibility, on the other side, partial differential equations are easier to handle from a mathematical point of view.

Agent based models are slightly more natural than differential approaches since they mimic more closely the nature of the real phenomenon. The proportion density of the CTL has a questionable existence since CTL are positioned into the systems and evolve stochasticly.

In this work, we aim to describe a possible interacting effect in the population of CTL due to chemotactism for a specific subpopulation of CTL. This phenomenon can be handled with stochastic particles without any difficulty, by the addition of a drift to the symmetric random walk described by (1). This leads to a re-inforced random walk whose simulation is simple and easy to reproduce or modify. By no means this chemotactism can be described so easily with PDE. Recent advances in this direction relies on Keller-Segel models (see for instance [16]) but these models are far from being trivially solved by PDE. Especially, these models need to be enhanced to implement a chemotactism towards a specific zone of the population of CTL. We are currently working on an improvement of a Keller-Segel type description of our setting with the view to obtain simpler equations.

## Materials and Methods

### Experimental parameters measurement

The mean velocity of CTL displacement (8.66 *μm*/*min*) was measured by time-lapse microscopy using a confocal laser-scanning microscope (either a Zeiss LSM-510 or a Zeiss LSM-710 microscope, Zeiss Germany). Measurements were performed on non-stimulated CTL that were free to move on poly-D-lysine coated LabTeck-chambers.

The mean time required for killing a melanoma cell (26 minutes) was estimated by measuring, in a significant number of CTL/target cell conjugates, the time occurring between the initial CTL/target cell contact and the beginning of blebbing in target cells, as reported in I. Caramhalo et al. by time-lapse confocal microscopy [14]. Experimental measurements of the mean time required for melanoma cell killing by CTL were employed to estimate *μ*.

Melanoma cell mean diameter (12.5 *μm*) was estimated on paraformaldehyde fixed melanoma cell spheroids using a confocal microscope.

The time required for melanoma cell division was measured in standard melanoma cell 2-D cultures by counting the cell number at fixed time intervals over a total period of three days.

Melanoma cell spheroids were generated using the ‘hanging-drop’ method. Briefly 1000 cells/25*μl* were seeded in the wells of a Terasaki culture plate. The plate was then inverted to form suspended droplets. The measurement of the diameter of the spheroids was performed using a Zeiss LSM-710 microscope using a 20x objective. The mean diameter of a spheroid was estimated by calculating the mean value of 4 diameters measured using the LSM software (Zeiss). To visualize the alive/dead fraction of cells in the spheroid at day 6 of culture the spheroids were incubated for 2 hours with 5nM Ethidium-1-HomoDimer (Eth-1-HD, red) and 1 *μM* calcein (green) at 37^*o*^ *C*. Staining was visualized using a Zeiss LSM-710 using and 20x ojective.

For the measurement of CTL/tumor cell cluster formation, HLA-A2^+^ melanoma cells (D10) were loaded for 2 hours at 37^*o*^ *C*, either with NLVPMVATV or VLAELVKQI (two different epitopes of the human cytomegalovirus protein pp65), washed and conjugated either with the CTL clone VLA-E2 or with the CTL clone NLV-2 as indicated in S4 Fig. legend. After 30 minutes co-culture the same number of CMFDA-loaded VLA-E2 CTL (loaded with CMFDA-green) were added. After additional 48 hours cells were analyzed using a confocal laser scanning-microscope (Leica SP8). z-stacks of confocal images were acquired and analyzed using the Image J software.

CTL were purified from peripheral blood of healthy donors and were kept in culture by periodic re-stimulation with irradiated peripheral blood mononuclear cells. Peripheral blood mononuclear cells (PBMC) were isolated from buffy coats of healthy donors obtained through the Établissement Français du Sang (EFS Midi-Pyrénées, Purpan University Hospital, Toulouse, France). Blood samples were collected and processed following standard ethical procedures (Helsinki protocol), after obtaining written informed consent from each donor and approval for this study by the local ethical committee (Comité de Protection des Personnes Sud-Ouest et Outremer II).

### Time and Scale calibration: grid definition

In our grid model the distance between two neighbor cells is fixed according to the mean melanoma cell size, which is denoted Δ. The value Δ = 12.5*μm* was measured by confocal microscopy. The time-step along our iterations is strongly related to the velocity of CTL since it is the time necessary for one CTL to move from its position to one of its 1-neighborhood. In order to obtain an immediate velocity of each CTL that corresponds to the one observed on real data, we fix the time-step *δ*_*t*_ to be the mean time necessary for one CTL to cover a distance that corresponds to Δ. Experimental measurements of the displacements of CTL establish a mean velocity of *v* = 8.66 *μ*m/min. We then choose *δ*_*t*_ = 1.44 min, which means that there is about 1 minute and 30 seconds between two consecutive iterations of the random walks.

### Estimation of *μ*: the inverse of the time required for killing one target cell

We compute an estimation of *μ* in an independent context of our stochastic dynamical model. Experimental measurement of the time required for melanoma cell killing by CTL were employed to compute *μ* estimation [13]. For a number *N* of observations, we observe several lysis duration ${\left(t_{lysis}^{i}\right)}_{i\in \{1,\ldots ,N\}}$, and standard estimation on exponential law yields an estimator of the rate ${\hat{\mu }}_{N}^{-1}$, which is the inverse of the mean time of lysis. To obtain the rate according to our time step *δ*_*t*_, we then compute ${\hat{\mu }}_{N}^{*}$ as

$$\begin{array}{c} \frac{1}{{\hat{\mu }}_{N}^{*}}=\frac{1}{{\hat{\mu }}_{N}/\delta_{t}}=N\;{\left(\sum_{i=1}^{N}t_{lysis}^{i}\right)}^{-1}\times \delta_{t}. \end{array}$$

### Estimation of *λ*: rate of division

The estimation of the division rate *λ* of tumor cells is slightly more complex than the estimation of *μ* owing to the nature of available biological observations. Measurements were performed in culture conditions in which tumor cells grew in 2-D without forming cellular spheroids. In these conditions, the replication of the tumor cells evolves without any geometric constraints. The number of cells was kept fixed at time 0 (denoted *n*_0_) and counted at time T so that we obtained this final number *n*_*T*_. Value of tumor cell growth obtained in N individual culture plates corresponds to the observation of $\left(n_{T}^{1},\ldots ,n_{T}^{N}\right)$.

The model can be seen as a Pure Birth Process (Yule-Furry process) from a probabilistic point of view: when one tumor cell is replicated, the population increases of one individual cell. If we denote *X*_*t*_ the number of cells at time *t*, the probability that {*X*_*t*_ = *n*} given by *P*_*n*_(*t*) = *P*(*X*_*t*_ = *n*) satisfies

$$\begin{array}{c} P_{n}\left(t+h\right)=P_{n}\left(t\right)\left(1-\lambda nh\right)+P_{n-1}\left(t\right)\left(n-1\right)\lambda h+o\left(h\right). \end{array}$$

Since the probability that one birth occurs during the interval [*t*, *t* + *h*], up to the condition {*X*_*t*_ = *n*}, is *nλh* + *o*(*h*). Then (1 − *nλh*) + *o*(*h*) is the probability that no division appears during [*t*, *t* + *h*] if {*X*_*t*_ = *n*}. Then (*P*_*n*_(*t*))_*t* ≥ 0,*n* ∈ ℕ_ satisfies the differential system

$$\begin{array}{c} P_{n}^{'}\left(t\right)=-n\lambda P_{n}\left(t\right)+\left(n-1\right)\lambda P_{n-1}\left(t\right). \end{array}$$ (2)

Since the starting number of cells is *n*_0_, we have *P*_*n*_0__(0) = 1, and for all *n* ≠ *n*_0_, *P*_*n*_(0) = 0. We remind the notation of the binomial coefficients $\left(\begin{array}{c} n\\ k \end{array}\right)$, whose valued are defined as

$$\begin{array}{c} \left(\begin{array}{c} n\\ k \end{array}\right)=\frac{n!}{(n-k)!k!}. \end{array}$$

According to this definition, One may check that the unique solution of (2) is given by

$$\begin{array}{c} \forall n\geq n_{0}\;P_{n}\left(t\right)=\left(\begin{array}{c} n-1\\ n-n_{0} \end{array}\right)e^{-\lambda n_{0}t}{\left(1-e^{-\lambda t}\right)}^{n-n_{0}}. \end{array}$$ (3)

Let us briefly recall the proof of (3), which is obtained by induction. In this view, we denote (*H*_*n*_0_+*k*_) the induction assumption for the integer *n*_0_ + *k* and remark that an equivalent formulation of (3) is

$$\begin{array}{c} \left(H_{n_{0}+k}\right):\;\forall k\geq 0,\;P_{n_{0}+k}\left(t\right)=\left(\begin{array}{c} n_{0}+k-1\\ k \end{array}\right)e^{-\lambda n_{0}t}{\left(1-e^{-\lambda t}\right)}^{k}=\left(\begin{array}{c} n_{0}+k-1\\ k \end{array}\right)e^{-\lambda (n_{0}+k)t}{\left(e^{\lambda t}-1\right)}^{k}. \end{array}$$ (4)

Let us remark that (2) implies that *P*_*n*_0__ satisfies $P_{n_{0}}^{\prime }(t)=-n_{0}\lambda P_{n_{0}}(t)$, whose solution is

$$\begin{array}{c} P_{n_{0}}\left(t\right)=e^{-n_{0}t}P_{n_{0}}\left(0\right)=e^{-n_{0}\lambda t}\left(=\left(\begin{array}{c} n_{0}-1\\ 0 \end{array}\right)e^{-\lambda (n_{0}+0)t}{\left(1-e^{-\lambda t}\right)}^{0}\right). \end{array}$$

This proves that (*H*_*n*_0__) is true. Now, assume that (*H*_*n*_0_+*k*−1_) holds and we want to establish (*H*_*n*_0_+*k*_). Remark that (2) leads to

$$\begin{array}{c} \forall t\geq 0\;P_{n_{0}+k}^{'}\left(t\right)+\left(n_{0}+k\right)\lambda P_{n_{0}}\left(t\right)=\left(n_{0}+k-1\right)\lambda P_{n_{0}+k-1}\left(t\right). \end{array}$$

From (*H*_*n*_0_+*k*−1_), we deduce that this last equation is equivalent to

$$\begin{array}{c} \frac{d\left(P_{n_{0}+k}\left(t\right)e^{\lambda (n_{0}+k)t}\right)}{dt}e^{-\lambda (n_{0}+k)t}=\lambda \left(n_{0}+k-1\right)C_{n_{0}+k-2}^{k-1}e^{-\lambda (n_{0}+k-1)t}{\left[e^{\lambda t}-1\right]}^{k-1}. \end{array}$$

As a consequence, we get

$$\begin{array}{ccc} P_{n_{0}+k}\left(t\right)e^{\lambda (n_{0}+k)t}-P_{n_{0}+k}\left(0\right) & = & \left(n_{0}+k-1\right)\left(\begin{array}{c} n_{0}+k-2\\ k-1 \end{array}\right)\int_{0}^{t}\lambda e^{\lambda s}{\left[e^{\lambda s}-1\right]}^{k-1}ds\\ & = & \left(n_{0}+k-1\right)\left(\begin{array}{c} n_{0}+k-2\\ k-1 \end{array}\right)\int_{0}^{t}{\left[e^{\lambda s}-1\right]}^{k-1}d\left(e^{\lambda s}\right)\\ & = & \left(n_{0}+k-1\right)\left(\begin{array}{c} n_{0}+k-2\\ k-1 \end{array}\right)\int_{1}^{e^{\lambda t}}{\left[u-1\right]}^{k-1}du\\ & = & \left(n_{0}+k-1\right)\left(\begin{array}{c} n_{0}+k-2\\ k-1 \end{array}\right)\frac{{\left[e^{\lambda t}-1\right]}^{k}}{k}\\ & = & \left(\begin{array}{c} n_{0}+k-1\\ k \end{array}\right){\left[e^{\lambda t}-1\right]}^{k} \end{array}$$

Since the population is initialized with *n*_0_ cells, *P*_*n*_0_+*k*_(0) = 0 and we deduce that (*H*_*n*_0_+*k*_) holds (see Equation (4)).

Hence, *X*_*t*_ follows a negative binomial distribution 𝓝𝓑(*n*_0_, *e* ^−*λt*^) initialised with *n*_0_ cells and a succeed parameter *e* ^−*λt*^.

We aim to estimate *λ* and by denoting *p* = *e* ^−*λT*^, we can use the Maximum Likelihood Estimation (M.L.E.) to compute ${\hat{p}}_{N}$, and then ${\hat{\lambda }}_{N}$. Using our observations $\left(n_{T}^{1},\ldots n_{T}^{N}\right)$, the log-likelihood is given by

$$\begin{array}{c} l\left(n_{0},p,n_{T}^{1},\ldots n_{T}^{N}\right)=\log\;\left(\prod_{i=1}^{n}P_{n_{T}^{i}}\left(T\right)\right). \end{array}$$

We use now (3) to obtain

$$\begin{array}{c} l\left(n_{0},p,n_{T}^{1},\ldots n_{T}^{N}\right)=\sum_{i=1}^{N}\log\left(\left(n_{T}^{i}+n_{0}-1\right)!\right)-\sum_{i=1}^{N}\log\left(n_{T}^{i}!\right)-N\log\left(\left(n_{0}-1\right)!\right)+Nn_{0}\log\left(p\right)+\sum_{i=1}^{N}n_{T}^{i}\log\left(1-p\right). \end{array}$$

We can optimize the last expression with respect to *p* to obtain that

$$\begin{array}{c} {\hat{p}}_{N}=\arg\max_{0\leq p\leq 1}\;l\left(n_{0},p,n_{T}^{1},\ldots n_{T}^{N}\right)=\frac{n_{0}}{n_{0}+\sum_{i=1}^{N}\frac{n_{i}^{T}}{N}}. \end{array}$$

Since we have set *p* = *e* ^−*λT*^, we deduce that ${\hat{\lambda }}_{N}$ is given by ${\hat{\lambda }}_{N}=T^{-1}\log\;\left(1+\sum_{i=1}^{N}\frac{n_{T}^{i}}{Nn_{0}}\right).$ As in the estimation of *μ*, to obtain the rate according to time step, we then compute ${\hat{\lambda }}_{N}^{*}={\hat{\lambda }}_{N}\times \delta t.$

### Estimation of *E*: thickness of the proliferative part of the nodule

The size of the proliferative part is an important feature for the growth of the tumor nodule. Hence, *E* should be carefully estimated: we assume in the sequel that *E* is constant over the time evolution of the nodule. In order to fix a plausible value of *E*, the global diameter of the nodule was observed over the time. We denote (*d*_*t*_)_0 ≤ *t* ≤ *T*_ such a diameter from the initial time *t* = 0 to the ending one *t* = *T* (see S3 Fig.) and design a mathematical study that describes the effect of *E* on the average evolution of the diameter of the nodule. The main difficulty to estimate *E* relies on the nature of our observations, that are dependent on the temporal evolution of the diameter of the nodule.

For the sake of simplicity, we assume here a circular structure of the tumor with an inner necrotic part, whose radius is ${\tilde{r}}_{t}$, and an outer proliferative part between ${\tilde{r}}_{t}$ and $r_{t}={\tilde{r}}_{t}+E$ (see S2 Fig.). The observed diameter is then *d*_*t*_ = 2*r*_*t*_. From the tumor growth model, the division of a tumoral cell yields an addition of a new cell in the proliferative part if the mother cell does not belong to the frontier with the necrotic part of the nodule (whose radius is ${\tilde{r}}_{t}$). If Γ_*t*_ is the average number of tumoral cells in the proliferative part, we then have

$$\begin{array}{c} \Gamma_{t+h}=\Gamma_{t}+\lambda \Gamma_{t}\times p_{\Delta ,E}, \end{array}$$ (5)

where *p*_Δ,*E*_ is the proportion of mother cell in the proliferative part that does not encounter the necrotic one. This probability can be approximated as soon as $\max(\Delta ,E)<<{\tilde{r}}_{t}$:

- The area of the cells that belong to the frontier of the necrotic part is given by
  $$\begin{array}{c} {\left({\tilde{r}}_{t}+\Delta \right)}^{2}-{\tilde{r}}_{t}^{2}∼2{\tilde{r}}_{t}\Delta . \end{array}$$
- The area of the proliferative part is given by
  $$\begin{array}{c} {\left({\tilde{r}}_{t}+E\right)}^{2}-{\tilde{r}}_{t}^{2}∼2{\tilde{r}}_{t}E. \end{array}$$

As a consequence,

$$\begin{array}{c} p_{\Delta ,E}=1-\frac{{\left({\tilde{r}}_{t}+\Delta \right)}^{2}-{\tilde{r}}_{t}^{2}}{{\left({\tilde{r}}_{t}+E\right)}^{2}-{\tilde{r}}_{t}^{2}}≃1-\frac{\Delta }{E}. \end{array}$$

If we denote $\tilde{\lambda }=\lambda (1-\frac{\Delta }{E})$, the solution of Equation (5) is

$$\begin{array}{c} \Gamma \left(t\right)=\Gamma \left(0\right)e^{\tilde{\lambda }t}. \end{array}$$ (6)

We can use the observed sequence (*d*_*t*_)_0 ≤ *t* ≤ *T*_ of the diameter of the nodule to obtain a second equation on Γ(*t*) since Γ(*t*) is related to *d*_*t*_ by the following formula:

$$\begin{array}{ccc} \Gamma (t) & = & \underset{\text{number}\;\text{of}\;\text{cells}\;\text{in}\;\text{the}\;\text{nodule}}{\underset{︸}{\frac{d_{t}^{2}}{\Delta^{2}}}}-\underset{\text{number}\;\text{of}\;\text{cells}\;\text{in}\;\text{the}\;\text{inert}\;\text{part}}{\underset{︸}{\frac{{\left(d_{t}/2-E\right)}^{2}}{{\left(\Delta /2\right)}^{2}}}} \end{array}$$ (7)

$$\begin{array}{ccc} & = & \frac{4E(d_{t}-E)}{\Delta^{2}}. \end{array}$$ (8)

Using this last equation at time *t* = 0 and *t* = *T*, and using (7) in (6), *E* should satisfy:

$$\begin{array}{ccc} \frac{4E(d_{t}-E)}{\Delta^{2}}=\frac{4E(d_{0}-E)}{\Delta^{2}}e^{\tilde{\lambda }t} & \Leftrightarrow & E=\frac{\Delta \lambda t}{\lambda t-\log\left(d_{t}-E\right)+\log\left(d_{0}-E\right)}. \end{array}$$ (9)

It is then possible to numerically solve this equation, but as we can only consider entire parts of cell sizes, we choose $E^{*}=\Delta \left\lceil \frac{E}{\Delta }\right\rceil$, where E* is the size of the proliferative part in our simulations. We obtained with our data E* = 2Δ. As a goodness of fit testing for our value E*, S4 Fig. compares the experimental observed diameter with the evolution of the theoretical diameter (*d*_*t*_)_0 ≤ *t* ≤ *T*_ obtained with (7) (for E* = Δ and E* = 2Δ: $d_{t}=\frac{\Delta^{2}\Gamma (t)}{4E}+E,$ for all 0 ≤ *t* ≤ *T*).

## Results

### Early productive CTL/tumor collisions determines CTL success in tumor eradication

Having established the basic parameters of the model, we employed numerical simulations to compute the probability of success of CTL in eradicating the tumor nodule without chemotactism, *i.e*. meaning that CTL motility is driven by a pure symmetric random walk. In a first approach, we investigated the number of collisions between CTL and tumor cells that were required to grant tumor eradication. For this analysis we considered a set of numerical simulations performed by varying the number of CTL ranging from 600 to 1100. This CTL range was chosen on the basis of initial observations indicating that 600 CTL exhibit a low chance of tumor eradication while 1100 exhibit a high chance of tumor eradication, within the defined size of the grid. Numerical simulations showed that, for CTL success, it was important that a minimum number of CTL/tumor cell productive collisions would occur during the early time points of CTL/tumor cell dynamic confrontation. More precisely, in our mathematical simulation lasting 72 hours, we computed *N*_*E*_, which was the number of collisions during the first 5 hours (defined as early collisions):

$$\begin{array}{c} N_{E}=\mathrm{card}\left\{\left(n,i\right)\;|\;z_{n}^{i}\;\text{hits}\;\text{the}\;\text{tumor}\;\text{nodule}\;\text{with}\;n\leq 5\;\text{hours}\right\} \end{array}$$

**Fig 2:**
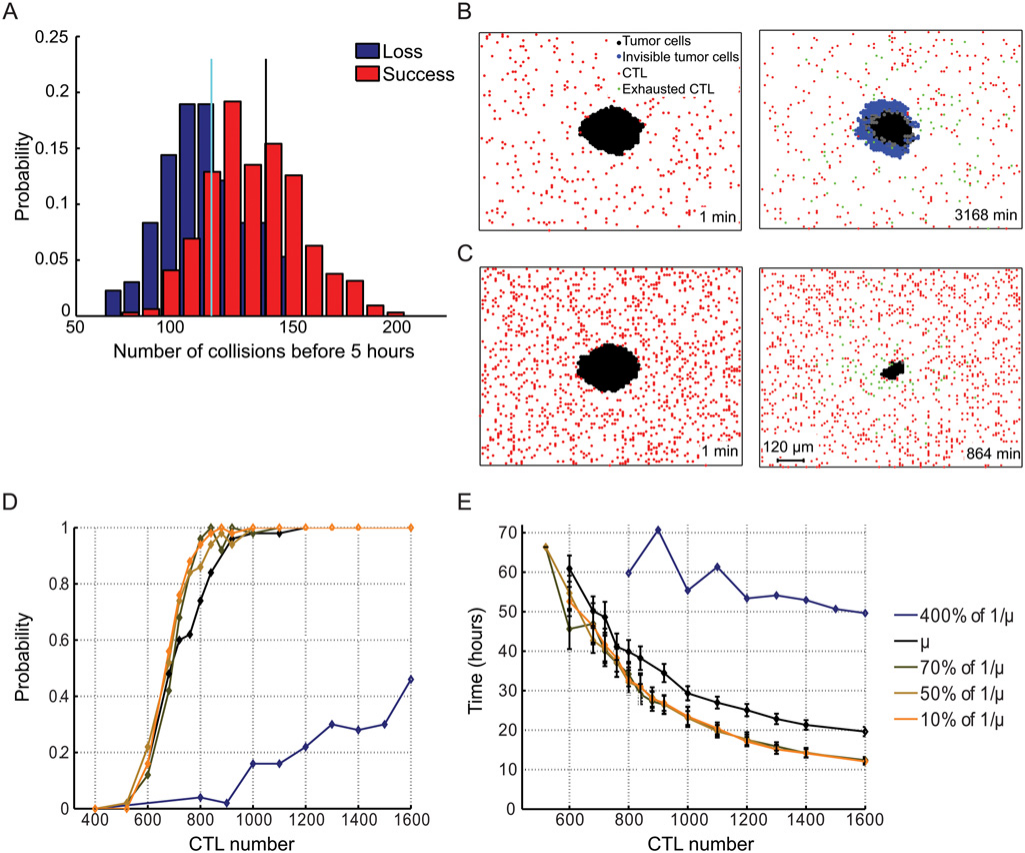
The probability of tumor nodule eradication increases with the increase of CTL number. (*A*) Empirical distribution of the number of early collisions (*N*_*E*_) (CTL killing during the first 5 hours) in the CTL loss scenario (blue) and its respective mean number of collisions (cyan bar). Same computations in the case of CTL victory scenario (red) and its respective mean number of early collisions (black bar). (*B*) Snapshots taken at times *t* = 1*min* and *t* = 3168*min* of the simulated interaction between 400 CTL and a growing tumor nodule (see S1 movie). A large number of tumor cells become invisible (blue). (*C*) Snapshots taken at times *t* = 1*min* and *t* = 864*min* of the simulated interaction between 1200 CTL and a growing tumor nodule. Invisible tumor cells have not been generated and the CTL population eradicates the tumor nodule. (*D*) Estimation of the victory’s probability of the CTL population in tumor nodule eradication (*P*_*S*_). Black line: estimated probability with respect to the number of CTL without any modification of the mean killing time 1/*μ*. Blue line: the mean killing time is augmented of 4 times. Other lines: same evolution when the mean killing time is decreased by 10%, 50% and 90%. (*E*) Mean time needed to eradicate the tumor nodule for the CTL population (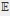
(*T*_*S*_)). Results are presented as means ± standard deviation of 50 numerical simulations. Black line: estimated time without any modification of the mean killing time (other lines: with increase or decrease of the mean killing time, as indicated in (*D*)).

Simulations showed that *N*_*E*_ was significantly larger in the case of CTL success than in the case of CTL incapacity to eradicate the tumor (Fig. 2*A*). Fig. 2*A* also shows that

- the average value of *N*_*E*_ corresponding to CTL inefficacy was 𝔼[*N*_*E*_] ≃ 112 collisions/5 hours
- the average value of *N*_*E*_ corresponding to tumor eradication was 𝔼[*N*_*E*_] ≃ 137 collisions/5 hours.

Taken together the above results indicate that in a cancer immunoediting scenario, the larger the number of early collisions *N*_*E*_ between CTL and target cells, the higher the probability of tumor eradication.

### Early productive collisions depend on CTL population size

In order to define the parameters that might ensure a sufficient number of early collisions leading to tumor eradication, we initially varied the size of the CTL population. CTL follow a pure symmetric random walk for their displacement. The number of CTL used in the simulations should not be considered as absolute, but it should be related to the dimension of the grid. We varied the CTL number for two reasons. First, because it has been previously described that the number of available killer cells is a crucial parameter in defining the success of a killer cell population over tumors [17]. Second, because it has been experimentally demonstrated that the capacity to eliminate tumor target cells increases with the size of the CTL population [14]. In line with these reported data, our numerical simulations showed that the probability of success in tumor nodule eradication was improved with the increase of CTL number while the mean time required for nodule eradication decreased (Fig. 2 *B*–*E* and S1 and S2 Movies). This probability of success is denoted below *P*_*S*_ and corresponds to the empirical probability of success of the CTL population over 50 Monte-Carlo runs. We observe that

- *P*_*S*_ ≥ 0.95 as soon as the CTL number is higher than 900 (for the estimated value of mean killing time $\frac{1}{\mu }$),
- *P*_*S*_ ≤ 0.15 when the CTL number is lower than 600 (for the estimated value of mean killing time $\frac{1}{\mu }$).

**Fig 3:**
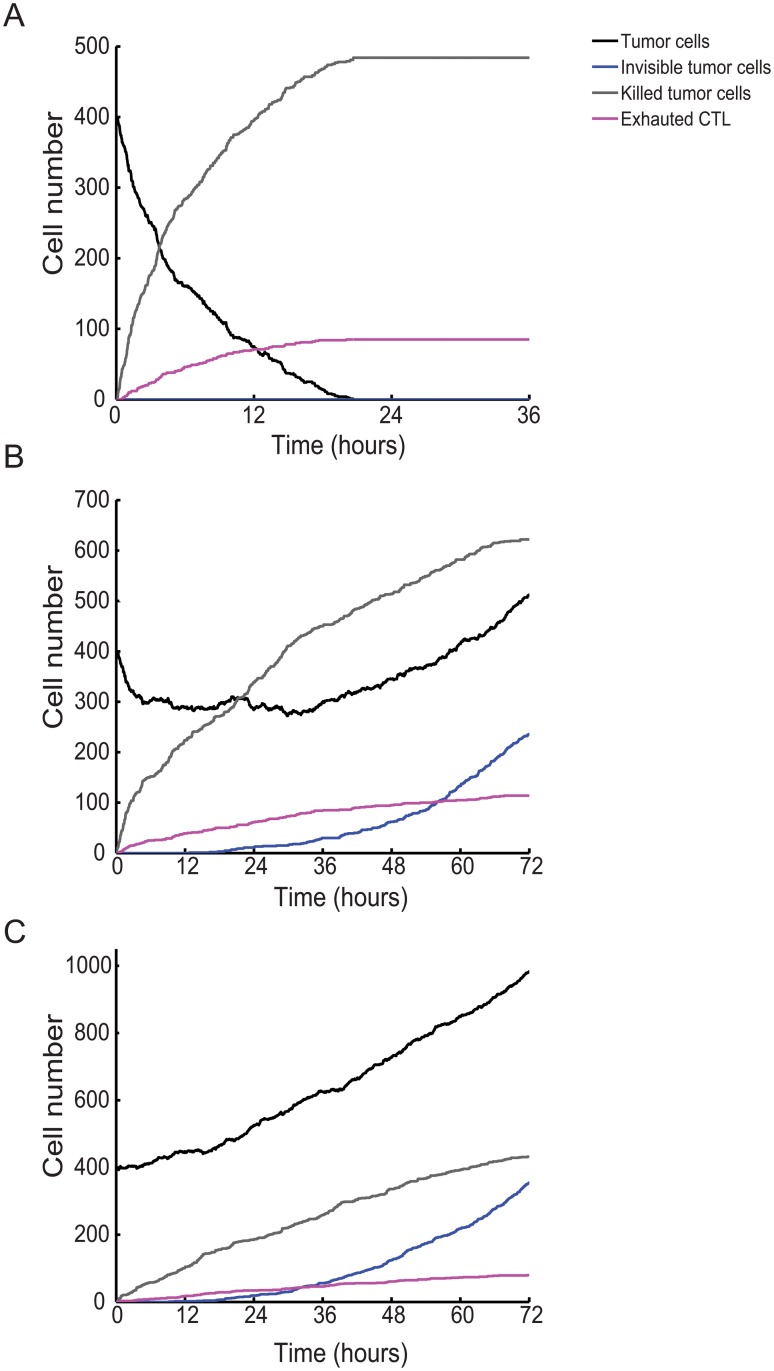
Evolution of the number of cells over time as a function of the initial CTL number. (A) 1200 CTL are present at the beginning of the simulation. (B) 400 CTL are present at the beginning of the simulation. (C) 200 CTL are present at the beginning of the simulation.

An alternative way to graphically represent the impact of CTL number on the probability of success in tumor nodule eradication is shown in Fig. 3 in which three simulations performed with high (1200, A), intermediate (400, B) and low (200, C) CTL number are presented. The plots represent the number of cells in the tumor nodule, the number of killed tumor cells, the number of exhausted CTL and the number of invisible tumor cells over time. They show that, while at high and low CTL numbers the balance between CTL success and tumor resistance is rapidly in favor of CTL or of tumor respectively (A and C), at intermediate cell numbers a more complex behavior is observed (B). A first phase in which CTL manage to reduce tumor size is followed by a period of equilibrium in which the size of tumor nodule remains stable. These phases are followed by a phase in which the number of tumor cells starts to grow in parallel with the stochastic generation of tumor cells not visible by CTL. Interestingly, these three phases in tumor/CTL confrontation observed at intermediate CTL number are reminiscent of the three phases described in cancer immunoediting: elimination, equilibrium and escape [6].

To investigate if the increase in the number of killer cells have an impact on the number of early CTL/target cell productive collisions, we measured the distribution of *N*_*E*_ (the number of collisions during the first 5 hours) under different conditions. This analysis showed that when the number of CTL was increased from 400 to 1600 a sharp increase of the productive early collisions was observed (S1 A Fig., compare black and cyan histograms, mean values of 𝔼(*N*_*E*_) = 85 collisions/5 hours, black versus 𝔼(*N*_*E*_) = 186 collisions/5 hours, cyan).

Taken together these results indicate that the CTL population size strongly affects CTL/tumor nodule early collisions and, in turn, tumor eradication.

### Early productive collisions do not depend on the time required for killing

We next investigated the impact that CTL time of killing (defined as the time required by a single CTL to annihilate a target cell) might have on tumor eradication (without chemotactism). To this end, numerical simulations in which the time required to kill target cells was reduced to less than 10% of the experimentally measured time were performed [14]. Interestingly, a sharp reduction of the time required for killing of individual tumor cells (down to 3 minutes from the initial 26 minutes) did not significantly affect neither the efficacy of tumor nodule eradication by CTL nor the mean time required for nodule eradication (Fig. 2 *D*–*E*). Conversely, when the time of killing was increased 4 times (up to about 2 hours) a clear effect on the efficacy of CTL-mediated cytotoxicity was observed. Indeed, the probability of success went down to *P*_*S*_ ≤ 0.25 (for 1200 CTL), as compared to *P*_*S*_ = 1 measured for a killing time of 26 minutes.

We next investigated whether early CTL/tumor nodule productive collisions would depend on the time required for killing. As shown in S1 B Fig., when the time required to kill a target cell was reduced of 30% or of 90% we observed a similar moderate increase in the number of initial productive collisions (*N*_*E*_), indicating that it is not possible to surpass an upper limit of productive collision number by modulating the time of killing. The mean collision value was 𝔼(*N*_*E*_) = 130 (instead of 85) when the time of killing was reduced of 30%. A similar mean collision value of about 130 was observed when the time of killing was reduced of 90%.

Taken together, the above results support the finding that the number of productive contacts between CTL and tumor cells is an important parameter influencing the success of a CTL population. They show that, while an augmentation of the time required for killing of individual target cells strongly affects CTL efficacy, the sharp reduction of the time required for target cell killing does not significantly affect the probability of CTL success in tumor eradication.

### CTL chemotactism towards scout cells having detected the tumor strongly augments early productive collisions

In the above-described numerical simulations the model postulated a scenario in which CTL are not preferentially directed towards the growing tumor and follow as symmetric random walk. In these conditions, only CTL stochasticly colliding with a cognate tumor cell exhibit cytotoxicity, thus only a low percentage of CTL participate to the killing. As a consequence, the CTL population, in its whole, mostly ignores the growing tumor. We now consider the situation of a self-interacting population of random walks influenced by chemotactism, as described in the model.

**Fig 4:**
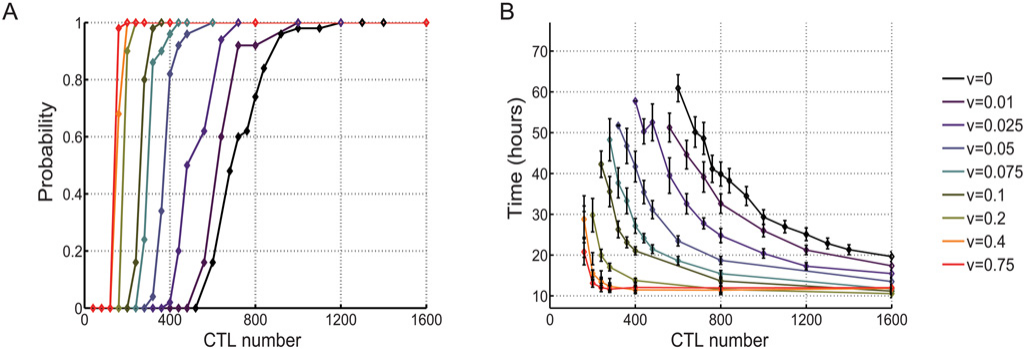
The capacity of scout CTL to attract other CTL strongly enhances tumor nodule eradication. (*A*) Evolution of the probability of the nodule eradication (*P*_*S*_) when an attraction strength is added to the (default) symmetric random walk dynamic, number going from 0.01 to 0.75 indicate increasing attraction. (*B*) Mean time of nodule eradication (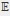
(*T*_*S*_)) when attraction is added to the default symmetric random walk dynamic of an increasing number of CTL. Results are represented as mean ± standard deviation of 50 numerical simulations.

Numerical simulations based on the above-described biased movement show that the probability of success in tumor nodule eradication increases with the increase of CTL attraction towards the scout cells. As shown in Fig. 4 and S3 Movie, with a relatively low number of CTL, the increase of the attraction of CTL towards scouts having detected the tumor nodule, strongly augments the probability of tumor nodule eradication. For instance for 400 CTL with no attraction there was no chance of success (see Fig. 4*A*), while a relatively moderate attraction of *ν* = 0.1 guarantees almost 100% probability of success. Lower attraction strengths (ranging between 0.01 to 0.075) still yielded significant impact on the probability of tumor eradication. Fig. 4*A* also shows that attraction reduces the range between the minimal number of CTL required for 100% eradication and success (exhibiting probability of tumor eradication equal to 1, denoted *N*_*S*_) and the maximal number of CTL failing in tumor eradication (exhibiting probability of tumor eradication equal to 0, denoted *N*_*F*_). For example, when *κ* = 5, we can show that:

- when *ν* = 0 (no attraction through chemotactism), the maximal failing number *N*_*F*_ ≃ 600 and the minimal success number *N*_*S*_ ≃ 1200, leading to *N*_*S*_ − *N*_*F*_ ≃ 600,
- when *ν* = 0.1, *N*_*F*_ ≃ 200 and *N*_*S*_ ≃ 350, leading to *N*_*S*_ − *N*_*F*_ ≃ 150.

These results indicate that the attraction “sharpens” the phase of transition in the number of CTL that are determinant for tumor eradication in the sense that the difference between *N*_*S*_ and *N*_*F*_ becomes smaller when the attraction strength *ν* is increased. Finally, the stronger the attraction *ν*, the earlier is the phase transition of the probability of CTL success.

- when *ν* = 0, *N*_*S*_ ≃ 1200,
- when *ν* = 0.1, *N*_*S*_ ≃ 350,
- when *ν* = 0.3, *N*_*S*_ ≃ 180.

The mean time required for nodule eradication (denoted *T*_*S*_) decreased accordingly (Fig. 4*B*). When we have a number of 400 CTL, we observe that:

- when *ν* = 0, the averaged time for tumor eradication is infinite (no tumor eradication),
- when *ν* = 0.1, 𝔼(*T*_*S*_) ≃ 21 hours,
- when *ν* = 0.3, 𝔼(*T*_*S*_) ≃ 12 hours.

To verify whether the increase of CTL attraction would result in an augmentation of productive early collisions, we measured the distribution of the number of collisions during the first 5 hours in conditions in which a moderate attraction was applied. As shown in S1 C Fig. the distribution of early collisions for a given number of CTL (400 CTL) was sharply increased in the presence of attraction (cyan histogram, 𝔼(*N*_*E*_) = 188 collisions/5 hours) when compared to the distribution of the same number of CTL with no attraction (black histogram, 𝔼(*N*_*E*_) = 85 collisions/5 hours). Taken together, the above results show that in a cancer immunoediting scenario the rapid recruitment of killer cells helps to reach a sufficient number of early productive collisions leading to tumor eradication.

## Discussion

In the present work, we applied mathematical modeling to dissect the multiparametric confrontation between CTL and a growing tumor nodule that undergoes immunoediting. We report that, for the given parameters, the success of a CTL population in tumor eradication strongly depends on the rate of CTL/tumor nodule productive collisions occurring during the initial period of CTL/tumor confrontation. In this view, we show that, when keeping the number of CTL constant, a bias in CTL motility inducing their attraction towards the tumor nodule is a major CTL functional parameter favoring CTL/tumor early collisions and tumor eradication.

It is well established that CTL are very efficient killer cells, that can rapidly annihilate target cells expressing a very small number of specific antigenic determinants on their surface [18, 19]. Moreover, CTL are known to be able to kill outnumbering target cells either simultaneously (via a mechanism named multiple killing [20]) or sequentially by bouncing from one target to another [21]. However, the rapidity and efficacy of CTL cytotoxic function does not apply to cancer immuno surveillance [22]. In the tumor microenvironment, the balance between CTL efficacy and tumor resistance to CTL attack might be in the favor of tumor escape because of: i) the progressive CTL inactivation in the immune-suppressive tumor microenvironment [5]; ii) the acquisition of resistance by tumor cells via the process of immunoediting [6]. Recent results show that these two processes are interconnected. The set up of multiple checkpoints in cancer microenvironment (such as regulatory T cell recruitment, expression of PDL-1, etc) aiming at inactivating CTL has been shown to be provoked by CTL themselves [10]. An additional important constraint to the capacity of CTL to eliminate tumors is the intrinsic resistancy of tumor cells to CTL attack. In a previous study we have shown that melanoma cells that have been previously loaded with viral antigens are recognized by virus-specific CTL and elicit their complete activation. Yet, under this condition melanoma cells exhibit an important resistance to CTL attack [14].

Although mathematical modeling of CTL attack of tumor cells has been previously performed [23, 24], a mathematical model that globally describes the whole cellular dynamics during tumor nodule growth and immunoediting is missing. In this work, we provide a stepping-stone to address this complex issue. We propose a precise stochastic dynamical system mostly based on experimentally measured parameters that allows to visualize and quantify the probability of success of the whole CTL population. We provide the unexpected observation that the decrease of the time required to kill a target cell by an individual CTL does not strongly improve the performance of the entire CTL population. This result can be explained by the fact that the time of the experimentally measured tumor cell duplication is much longer than the mean time of killing. Since these two time frames do not belong to the same scale, reducing the killing time has only a minor impact on tumor eradication. This result, however does not exclude the possibility that, if the rate of tumor cell duplication would become exceptionally high, a quicker killing time would be relevant for the success of the CTL population.

When we strongly increased the time required to kill target cells, this manipulation had a strong impact on CTL efficacy because tumor cell duplication and time required for killing get closer. These results are somehow expected and easy to explain on the basis of our knowledge of the biology of tumor nodule growth and CTL-mediated cytotoxicity. Yet, these results might be interesting to keep in mind in a scenario of anti-tumor immunotherapy. In the context of an *in vivo* confrontation between CTL and tumor cells, variations within a scale of minutes of the time required for killing should not strongly affect the success of an immunotherapy. Thus therapeutical strategies aiming at reducing the time required for killing might not elicit a substantial clinical improvement.

Conversely, a major obstacle to an efficient immunotherapy is the generation of tumor cell variants enduring for an extremely long time CTL attack or even becoming completely resistant to CTL-mediated cytotoxicity. Thus, successful immunotherapies are likely to be those avoiding or anticipating the generation of such variants by eradicating the tumor before substantial immunoediting.

Our numerical simulations show that the initial rate of productive collisions between CTL and tumor nodules is a key parameter in tumor eradication. This observation is by itself not fully surprising since it has been previously shown by both mathematical modeling [25] and experimental approaches [17] that increasing the ratio between effectors and target cells augments cytotoxicity. Nevertheless, our results allow to illustrate that, in a context of cancer immunoediting, a large number of CTL succeeds in tumor eradication because CTL can make a large number of early contacts with target cells. As a consequence, tumor cells are annihilated before they can accumulate mutations making them progressively resistant or even invisible to CTL. Numerical simulations show that biasing CTL random walk towards the tumor strongly increase the probability of CTL success. This striking success is mainly due to the augmentation of the number of early collisions avoiding immunoediting. It is tempting to speculate that this scenario might be amplified in a 3-D situation, since 3-D stochastic collisions are likely to be rare (symmetric random walk is null recurrent in 2-D and transient for a larger dimensions).

Numerical simulations thus reveal an interesting and previously undescribed critical behavior of CTL in tumor eradication: guiding CTL trajectories towards tumor nodules is crucial for efficient detection of a tumor by the CTL population as a whole.

CTL motility biasing towards scout cells has a biological justification in that CTL are known to store into cytoplasmic vesicles the CTL-attracting chemokine CCL5 and to release it rapidly after TCR stimulation [7]. Moreover, our experimental results indicate that localized activation of CTL at the contact site with tumor cell clusters enhances the adhesion of antigen-specific and non-specific CTL to the same cluster (S4 Fig.), suggesting a scouting function of activated CTL. Our observations are in line with results obtained in an *in vivo* model of CTL-mediated immunity against Plasmodium yoelii [26]. Taken together, these observations suggest that there is a natural aptitude of CTL to scout each other. It would be therefore interesting to potentiate this function in the context of anti-tumor therapies aiming at accelerating CTL based-responses against tumors.

A thorough characterization of the observed sharp phase of transition in CTL responses when attraction is present, is relevant to better understand the collective functional behavior of CTL populations. The capacity of individual T cells to provide all-or-nothing responses has been thoroughly documented both at the molecular and at the cellular level [27, 28] and has been illustrated by mathematical modeling of TCR-associated signaling pathways [29]. Here we show that, in conditions of CTL directed migration towards the tumor, the entire CTL population responds more rapidly and efficiently for tumor eradication. We thus extend the notion of all-or-nothing T cell responses from the individual T cell level to the entire T cell population for a given complex response. Such behavior of a T cell population reinforces the analogy between a whole T cell population and a sensory organ.

It has been previously proposed that T cells might be viewed as a type of sensory cells and that a multitude of T lymphocytes, with different specificities, can behave as sensory organs [30]. In the present work we extend this notion by proposing that a tumor-specific CTL population can be viewed as a sensory organ that, in its whole, ignores the developing tumor nodule because of a perception bias. In cognitive sciences, the biased competition theory of perception postulates that mental processes can bias the visual perception of objects by prioritizing one object in the visual field instead of another. By analogy to the visual cortex, the CTL population analyzed in this study would be naturally biased to detect pathogens presented by professional antigen presenting cells and would therefore tend to ignore self-antigens expressed by indolently growing tumor cells. The addition of a bias in CTL displacement compensates for this perception defect and makes the entire CTL population more successful in tumor detection and eradication.

An interesting question is whether the chemotactic movement of CTL towards the tumor nodule might synergize with other parameters that could positively influence CTL success in tumor eradication. To this end, we augmented the number of target cells killed by each CTL before getting exhausted. For 400 CTL, if we assumed that a CTL could kill 10 target cells before becoming exhausted (instead of 5 target cells), we observed that the probability to eradicate the nodule increased from 0 to 0.8 in conditions in which no chemotactism was present. Addition of chemotactism (*ν* = 1) increased this probability to 1. This apparent synergy was even more evident for 200 CTL. In this case, the probability to eradicate the nodule reached 0.9 in the presence of both chemotactism and killing of 10 target cells, while it was 0 when only one of the two conditions was present.

An interesting aspect of our approach is that the mathematical model and the biased competition theory of CTL migration can be extended to different predator/prey dynamical systems as well as colonies of self-interacting animals (ants, sheep, etc.) in which the success of the population depends on biased displacement and self-interacting behaviors of particles. The mathematical model raises several challenging questions such as the theoretical estimation of the probability of success (with respect to *ν*, *μ*, *λ*, etc) and the mathematical characterization of the phase transition observed in our simulations with increasing number of CTL in conditions in which CTL displacement is biased towards the tumor nodule. In the context of tumor immunology the described model should be instrumental to mimic the dynamic confrontation between CTL and tumor cells in other cancer diseases. Numerical simulations can integrate experimental data coming from different cellular models in order to define the parameters that, in each model, are more likely to shift the balance between CTL efficacy and tumor cell resistance in favor of CTL.

In conclusion, we propose a biased competition theory of CTL function in which the trajectories of individual CTL are guided by scout siblings resulting in efficient detection of tumor cells. Such a mechanism favors the success of the entire CTL population that, like a sensory organ, responds to biased stimuli for efficient signal/noise discrimination. The capacity of individual CTL to kill multiple targets either simultaneously or sequentially synergizes with CTL biased displacement in tumor eradication. Our results suggest that, to be successful, therapeutic strategies based on CTL adoptive transfer should aim at potentiating these two synergistic functions of CTL in an attempt to prevent CTL exhaustion and cancer immunoediting.

## Supporting Information

S1 Fig Distribution of the number of early collisions as a function of different parameters. (*A*) Empirical distribution of the number of early collisions (*N*_*E*_) (CTL killing during the first 5 hours) with a low number of CTL in black (400 CTLs) and a large number of CTLs in cyan (1600 CTLs). (*B*) Empirical distribution with an experimental measured killing time *μ* ^−1^ in black. Empirical distribution with a time decreased of 30% in cyan, and of 90% in green. (*C*) Empirical distribution with no attraction (*ν* = 0) in black and with attraction (*ν* = 0.3) in cyan.
(EPS)

S2 Fig Schematic representation of the inert and proliferative part of a tumor nodule. The proliferative part is of size *E*, each cell has a size Δ.
(EPS)

S3 Fig Measurement of the diameter of tumor nodules over time. (*A*) and (*B*) Experimental results. (*C*) Comparison of experimental measurements of the diameter of nodules over time (black line) and simulated measurement of nodules over time with *E* = 2 tumor cells (magenta) and with *E* = 1 tumor cell (blue).
(EPS)

S4 Fig Activation of CTL at the contact site with tumor cell clusters enhances the adhesion of antigen specific and non-specific CTL. (A) HLA-A2^+^ melanoma cells (D10) were loaded with the human cytomegalovirus protein pp65 peptide NLVPMVATV. A CTL clone (VLA-E2) specific for a different epitope of the human cytomegalovirus protein pp65 peptide: VLAELVKQI was conjugated by centrifugation with the D10 cells (unstained, not-green). After 30 minutes the same CTL (green) were added to the culture. Panel A shows typical images of CTL interacting with target cells loaded with an irrelevant antigenic peptide. (B) D10 cells were pulsed with the human cytomegalovirus protein pp65 peptide VLAELVKQI. The CTL clone VLA-E2 specific for this peptide was conjugated by centrifugation with the D10 cells (unstained). After 30 minutes the same CTL (green) were added to the culture. Panel B shows typical images of CTL interacting with target cells loaded with the specific antigenic peptide. (C) D10 cells were pulsed with the human cytomegalovirus protein pp65 peptide NLVPMVATV. The CTL clone (NLV-2) specific for the NLVPMVATV peptide was conjugated by centrifugation with D10 cells (unstained). After 30 minutes VLA-E2 CTL that are non-specific for this peptide (green) were added to the culture. Panel C shows the non-specific CTL VLA-E2 adhering to clusters formed by the specific CTL (NLV-2) with their target cells. z-stacks were acquired using a confocal laser-scanning microscope after 48 hours co-culture. Panels show the sum of the z-stack images. Data are from one representative experiment out of three.
(TIF)

S1 Movie The movie represents a mathematical simulation of the interaction between CTL and a tumor nodule undergoing immunoediting. The CTL/tumor cell ratio was 1:1. Black dots tumor cells, grey dots resistant tumor cells, blue dots invisible tumor cells, red dots CTL and green dots exhausted CTL. See Fig. 2 of the main text.
(AVI)

S2 Movie The movie represents a mathematical simulation of the interaction between CTL and a tumor nodule undergoing immunoediting. The CTL/tumor cell ratio was 3:1. Black dots tumor cells, grey dots resistant tumor cells, red dots CTL and green dots exhausted CTL. See Fig. 2 of the main text.
(AVI)

S3 Movie The movie represents a mathematical simulation of the interaction between CTL and a tumor nodule undergoing immunoediting. The CTL/tumor cell ratio was 1:1. An attraction toward the tumor nodule with a strength of 0.3 is applied. Black dots tumor cells, grey dots resistant tumor cells, red dots CTL and green dots exhausted CTL. See Fig. 4 of the main text.
(AVI)

S1 Table The Table shows a list of the parameters used in the model. (PDF)
