# Supplementary figures and images for: A Biased Competition Theory of Cytotoxic T Lymphocyte Interaction with Tumor Nodules
Source: PLoS One. 2015 Mar 27;10(3):e0120053. doi: 10.1371/journal.pone.0120053 (PMC4376944; doi:10.1371/journal.pone.0120053)

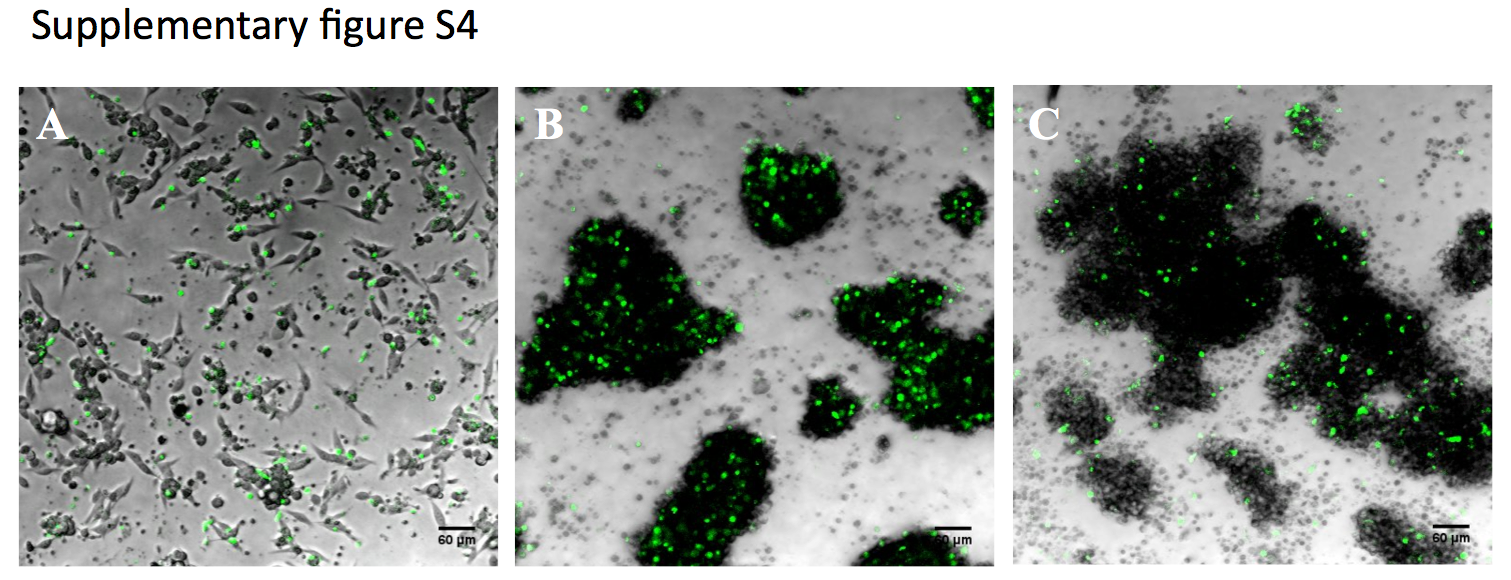

Supplement: S4 Fig — (A) HLA-A2+ melanoma cells (D10) were loaded with the human cytomegalovirus protein pp65 peptide NLVPMVATV. A CTL clone (VLA-E2) specific for a different epitope of the human cytomegalovirus protein pp65 peptide: VLAELVKQI was conjugated by centrifugation with the D10 cells (unstained, not-green). After 30 minutes the same CTL (green) were added to the culture. Panel A shows typical images of CTL interacting with target cells loaded with an irrelevant antigenic peptide. (B) D10 cells were pulsed with the human cytomegalovirus protein pp65 peptide VLAELVKQI. The CTL clone VLA-E2 specific for this peptide was conjugated by centrifugation with the D10 cells (unstained). After 30 minutes the same CTL (green) were added to the culture. Panel B shows typical images of CTL interacting with target cells loaded with the specific antigenic peptide. (C) D10 cells were pulsed with the human cytomegalovirus protein pp65 peptide NLVPMVATV. The CTL clone (NLV-2) specific for the NLVPMVATV peptide was conjugated by centrifugation with D10 cells (unstained). After 30 minutes VLA-E2 CTL that are non-specific for this peptide (green) were added to the culture. Panel C shows the non-specific CTL VLA-E2 adhering to clusters formed by the specific CTL (NLV-2) with their target cells. z-stacks were acquired using a confocal laser-scanning microscope after 48 hours co-culture. Panels show the sum of the z-stack images. Data are from one representative experiment out of three. (TIF) [file pone.0120053.s004.tif]
