# Supplementary material for: A Biased Competition Theory of Cytotoxic T Lymphocyte Interaction with Tumor Nodules
Source: PLoS One. 2015 Mar 27;10(3):e0120053. doi: 10.1371/journal.pone.0120053 (PMC4376944; doi:10.1371/journal.pone.0120053)
Supplement: S1 Table — (PDF) [file pone.0120053.s008.pdf]

Supporting Information S1 Table.

	parameter	description	value
CTL	$v^{\star}$	CTL displacement velocity	$8.66\mu m/min$
	$\mu^{\star o}$	Inverse of the time required for killing one target cell	$0.038 min^{-1}$
	$\kappa^{\diamond}$	Number of tumor cells killed by a single CTL	5
	N^{o}_{CTL}	CTL number at the beginning of the simulation	
Melanoma Nodule	$\Delta^{\star}$	Melanoma cell diameter	$12.5\mu m$
	$L^{\diamond}$	Melanoma nodule diameter at the beginning of the simulation	$300\mu m$
	$\lambda^{\star}$	Inverse of the time required for tumor cell division	$0.001 min^{-1}$
	$E^{\star}$	Thickness of the proliferative shell of the tumor mass	2Δ
	$p^{\diamond}_{res}$	Probability of a tumor cell to become more resistant to CTL attack at each division	$\frac{1}{2}$
	$p^{\diamond}_{inv}$	Probability of a tumor cell to become "invisible" to CTL at each division	$\frac{1}{2}\left(\frac{1}{2}-\frac{1}{res}\right)$
Motility Biased	$D^{\diamond}$	Maximal distance allowed for CTL attraction (if distance between CTL and tumor mass $\geq \sqrt{D}$, CTL are not attracted)	$350\mu m$
	$K^{\diamond}$	Number of scout CTL generating a maximal attraction	10
	ν^{o}	Maximal level of attraction	

S1 Table. Parameters used in simulations.* estimated parameters, ^{o} varying parameters, $^{\diamond}$ arbitrary parameters.
